# Around the Hospitals

**Published:** 1894-02-17

**Authors:** 


### Around the Hospitals.

Notice to the Managers of Institutions.—Special space is now reserved for the insertion of notices of hospital meetings and festivals. The Editor requests that all notices may reach The Hospital Office, 428, Strand, at least one week before the date of each meeting.

**The Dumfries Infirmary.**—There are one or two special features of interest in the annual report and annual meeting of this institution. The report reveals a method of financing a hospital receiving patients from many districts which might with advantage be more widely adopted. In the perusal of the many records of institutions affording medical relief throughout the kingdom it is constantly forced upon our notice that, although a hospital may be most largely patronised by outlying districts, the responsibility of maintenance seems only properly recognised by the vicinity in which it is situated. In the case of the Dumfries Infirmary, distinct sums are set apart by the surrounding districts sending patients for treatment. A definite arrangement has, moreover, been recently made, fixing the grants received at £6 per bed. This is a feature in the van of progress; one step further forward was made at the annual meeting. This was the admission of children for medical treatment as young as four years of age, seven being the previous limit. It appears that the innovation is regarded as experimental, the rule only holding good during one year's trial. The question of a children's ward was brought up, but negatived on the score of expense, a mistaken conclusion, as we regard it, seeing that where children's wards exist a larger amount of interest is always attracted towards a hospital, and there is very little difficulty in securing endowed cots, and the small extra expenses of administration such an arrangement entails. Visitors to a hospital should not be regarded as an objection, as was suggested at the meeting. It is invariably to be seen that where visitors are welcomed funds flow in also. Dumfries Infirmary is undoubtedly flourishing, and we trust that at the next annual meeting the scheme for a children's ward will receive wise encouragement, in the interests of the institution and the patients.

**The Dover Hospital.**—It is not a very encouraging fact when a hospital finds the work it is effecting doubled during a certain period whilst the subscriptions remain almost stationary. Such is the case at the Dover Hospital. Naturally the increased expenditure has entailed debt, and not too soon the authorities of the institution have arisen to the fact that some strenuous effort to alter this condition of affairs must be made. It is therefore intended to institute a house-to-house canvass. It would seem hardly necessary to state in the local press that subscriptions are not limited to one guinea, but so the authorities of the Dover Hospital seem to think. Perhaps they know the resources of the neighbourhood better than we do, yet we venture to hope that the liberal response the appeal will receive will make such a clause unnecessary on any future occasion.

